# Pharmacokinetic Study of Withanosides and Withanolides from *Withania somnifera* Using Ultra-High Performance Liquid Chromatography-Tandem Mass Spectrometry (UHPLC-MS/MS)

**DOI:** 10.3390/molecules27051476

**Published:** 2022-02-22

**Authors:** Siddharth J. Modi, Anshuly Tiwari, Chetana Ghule, Sandeep Pawar, Ganesh Saste, Shubham Jagtap, Ruchi Singh, Amol Deshmukh, Aboli Girme, Lal Hingorani

**Affiliations:** 1 Analytical Development and Innovation Center, Pharmanza Herbal Pvt. Ltd., Anand 388435, Gujarat, India; rdbiotech@pharmanza.com (S.J.M.); bioanalysis@pharmanza.com (A.T.); adic@pharmanza.com (C.G.); lcms@pharmanzaherbals.com (S.P.); ard@pharmanzaherbals.com (G.S.); hplc2@pharmanza.com (S.J.); lal@pharmanzaherbals.com (L.H.); 2 New Product Development Department, Pharmanza Herbal Pvt. Ltd., Anand 388435, Gujarat, India; rdm@pharmanzaherbals.com; 3 Clinical Research and Intellectual Property Rights, Pharmanza Herbal Pvt. Ltd., Anand 388435, Gujarat, India; rd@pharmanzaherbals.com

**Keywords:** nutraceuticals, *Withania somnifera*, pharmacokinetics, bioanalysis, ADMET

## Abstract

*Withania somnifera* is a traditional Indian herb described under the ‘Rasayana’ class in Ayurveda, which gained immense popularity as a dietary supplement in the USA, Europe, Asia, and the Indian domestic market. Despite enormous research on the pharmacological effect of withanosides and withanolides, bioanalytical method development and pharmacokinetics remained challenging and unexplored for these constituents due to isomeric and isobaric characteristics. In current research work, molecular descriptors, pharmacokinetic, and toxicity prediction (ADMET) of these constituents were performed using Molinspiration and admetSAR tools. A rapid, selective, and reproducible bioanalytical method was developed and validated for seven withanosides and withanolides as per USFDA/EMA guidelines, further applied to determine pharmacokinetic parameters of *Withania somnifera* root extract (WSE) constituents in male *Sprague Dawley* rats at a dose of 500 mg/kg. Additionally, an *ex vivo* permeability study was carried out to explore the absorption pattern of withanosides and withanolides from the intestinal lumen. In silico, ADMET revealed oral bioavailability of withanosides and withanolides following Lipinski’s rules of five with significant absorption from the gastrointestinal tract and the ability to cross the blood-brain barrier. Upon oral administration of WSE, C*_max_* was found to be 13.833 ± 3.727, 124.415 ± 64.932, 57.536 ± 7.523, and 7.283 ± 3.341 ng/mL for withanoside IV, withaferin A, 12-Deoxy-withastramonolide, and withanolide A, respectively, with T*_max_* of 0.750 ± 0.000, 0.250 ± 0.000, 0.291 ± 0.102, and 0.333 ± 0.129 h. Moreover, at a given dose, withanoside V, withanolide B, and withanone were detected in plasma; however, the concentration of these constituents was found below LLOQ. Thus, these four major withanoside and withanolides were quantified in plasma supported by *ex vivo* permeation data exhibiting a time-dependent absorption of withanosides and withanolides across the intestinal barrier. These composite findings provide insights to design a clinical trial of WSE as a potent nutraceutical.

## 1. Introduction

Herbal nutraceuticals and functional foods have gained worldwide popularity since the last decade due to increasing awareness about health among consumers and a steady increase in life expectancy [1]. *Withania somnifera* (Ashwagandha), also known as ‘Indian Winter Cherry,’ is a valuable herb used as ‘Rasayana’ to promote a youthful state for physical and mental health for over 3000 years [2,3,4]. The National Center for Complementary and Alternative Medicines (NCCAM) of the US National Institute of Health has recently declared ‘Ashwagandha’ a high-priority topic for mechanistic research in humans [5]. The recent report published states total sales of $13.7 million for *Withania somnifera* in the natural retail market along with it being the third highest-selling product (with 45.2% sale growth; reached $10.8 million) after cannabidiol (CBD) and elderberry [6]. Roots of *Withania somnifera* have been used to prepare a tonic that enhances longevity, revitalizes the body, arrests the aging process, and augments a defense against dreadful diseases, such as cancer, Alzheimer’s, and epilepsy [7,8]. It has diversified pharmacological activities, including adaptogenic, anti-inflammatory, anti-stress, antioxidant, hepatoprotective, immunomodulatory, memory enhancer, neuroprotector, etc. [9,10,11,12,13,14,15].

**Figure 1:**
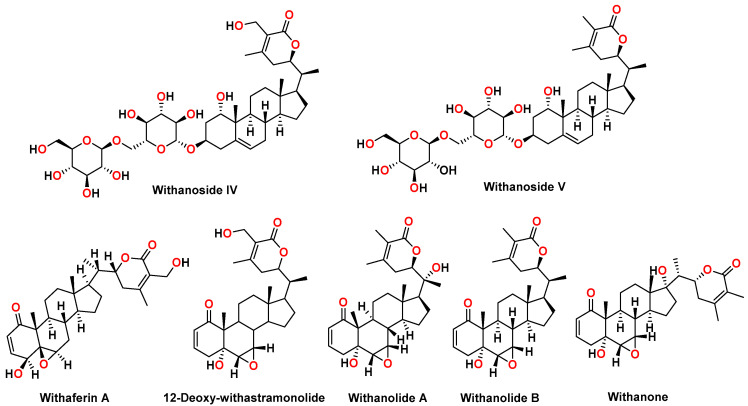
Chemical structures of withanosides and withanolides from *Withania somnifera* root extract: withanoside IV, withanoside V, withaferin A, 12-Deoxy-withastramonolide, withanolide A, withanolide B, and withanone.

*Withania* species consist of chemical constituents from diverse chemical classes, such as steroidal lactones, alkaloids, flavonoids, and tannins. More than 40 withanolides, 12 alkaloids, and numerous sitoindosides (withanolides consisting of a glucose molecule at C27) have been extracted and isolated from roots, berries, and aerial parts *Withania somnifera*. Different alkaloids, such as withanine, withananine, pseudowithanine, somniferine, and somniferinine can be isolated from the plant parts, specifically the leaves. However, the traditional Indian system of medicine called “Ayurveda” recommends using *Withania somnifera* roots for therapeutic purposes and internal administration. The chemical constituents of roots include withaferin A, withanolide A, withanolide B, 12-Deoxy-withastramonolide, and withanosides. Structures of withanolides and withanosides quantified from *Withania somnifera* root extract (WSE) is depicted in Figure 1.

Due to the complexity in the characterization of chemical constituents of medicinal plants, establishing pharmacokinetic and pharmacodynamic (PK/PD) relationships remained a constant challenge. Subsequently, the characterization of absorbed compounds, pharmacokinetics, tissue distribution, and elimination pattern of constituents upon oral administration from the body is of great interest [16,17,18]. This collective data provides a critical endpoint to determine pharmacological and clinical efficacy, resulting in very little information on how the body behaves with the drug/extract ingested. Amongst chemical constituents, comparative and/or synergistic absorption, distribution, metabolism, and excretion (ADME), differentiating factors can be observed when the whole extract is given to an animal/human compared to the individual constituent.

Although, chemical constituents of *Withania somnifera* present good and relatable biological activities in pre-clinical and clinical studies. However, the route of administration, dosage, pharmacokinetics, and biopharmaceutical profile are unexplored. The preceding information was used to standardize bioactive withanosides and withanolides of WSE. Later, an *in silico* absorption, distribution, metabolism, excretion, toxicity (ADMET), and molecular properties prediction of these withanosides and withanolides was carried out using admetSAR online tools. Further, a validated bioanalytical method for simultaneous estimation of withanoside IV, withanoside V, withaferin A, 12-Deoxy-withastramonolide, withanolide A, withanolide B, and withanone in plasma was developed using UHPLC-MS/MS. This method was applied to determine the pharmacokinetic parameters of withanosides and withanolides of WSE in rat plasma. Additionally, the permeability characteristic of WSE and its constituents was estimated using an *ex vivo* permeability model. Therefore, the results of these studies could be used to validate clinical trials in human volunteers. 

## 2. Results and Discussion

### 2.1. In Silico Molecular Properties and Absorption, Distribution, Metabolism, Excretion, and Toxicity (ADMET) Prediction 

Molecular descriptors of WSE constituents were determined for Lipinski’s rule of five (ROF). The molecular weight of the constituents was found in the range of 450–500 g/mol except for withanoside IV (782.92 g/mol) and withanoside V (766.92 g/mol). Molecular weight is an essential aspect concerning the biological action because as the molecular weight increases, the bulkiness of the compounds also increases correspondingly, which in turn affects the drug action (Table 1).

Lipophilicity (Log *p* value) and topological polar surface area (TPSA) are the two significant factors affecting the permeability of the compounds and determining oral bioavailability. TPSA can be defined as calculating the total surface area occupied by oxygen atoms, nitrogen atoms, and hydrogen atoms attached to these molecules. This points out a direct relation of the potential of hydrogen bonding with the TPSA value of the compound. The compounds having ≤140 Å TPSA value and rotational bonds ≤ 10 are more likely to have good bioavailability because rotational bonds give flexibility to the compound that can easily interact with specific rigid binding areas. Remarkably, here, five constituents of WSE demonstrated TPSA values under the specified limit.

In contrast, withanoside IV and withanoside V, showed higher TPSA values, which might be due to the structural diversity of the constituents in the extract. Subsequently, the Log *p* value of these constituents was calculated by Molinspiration software, where six constituents demonstrated a Log *p* value less than five. Withanolide B has a higher Log *p* value, which does not follow Lipinski’s rules of five. 

Drug solubility is one of the critical parameters that affect pharmacokinetics and pharmacodynamics, right from the administration, absorption into the systemic circulation, movement in the blood, and excretion. Here, ADMET prediction was carried out using the online dataset admetSAR. Permeability through various biological barriers, i.e., the blood-brain barrier (BBB), human intestinal absorption (HIA), human colorectal adenocarcinoma cell (Caco-2) permeability, and renal organic cation transport, was calculated. The ADMET characteristics majorly represented the probability to predict the certainty of the characteristic between 0–1 that gives an idea about the pharmacokinetics of the drug candidate through the prediction database. All the seven constituents of WSE can be absorbed from the intestine. Based on the results, five constituents, namely withaferin A, 12-Deoxy-withastramonolide, withanolide A, withanolide B, and withanone, might cross the BBB with the probability of >0.8, while withanoside IV and withanoside V could not cross the BBB. The *in silico* study indicates that withaferin A and 12-Deoxy-withastramonolide have the maximum oral bioavailability in the systemic circulation, supported by an *in vivo* pharmacokinetic study. The cytochrome enzymes are involved in drug metabolism for elimination and/or biotransformation. Prominent drug-drug interactions are reported due to the selective activation or inhibition of the cytochrome P450 (CYP) enzymes. Therefore, there is a possibility that the drug might be accumulated to a toxic level due to inhibition of CYP enzymes or rapidly excreted due to activation of CYP microsomal enzymes. Here, withanosides and withanolides could act as a substrate for the CYP450 3A4 enzyme while a non-substrate for CYP450 2C9 and CYP450 2D6 enzymes. Further, the results indicate that constituents of WSE were non-inhibitors for CYP450 1A2, CYP450 2C9, CYP450 2D6, CYP450 2C19, and CYP450 3A4 enzymes. A non-inhibitor of CYP450 suggests that the constituents might not hamper the biotransformation of drugs metabolized by the CYP450 enzyme. In pharmacology, P-glycoproteins are the major reason for drug resistance or making the cell less susceptible to the drugs. They are mainly involved in the efflux and activation of P-glycoprotein (P-gp), which would increase the drug’s efflux and create drug concentration below the minimum required concentration, leading to therapeutic failures. Interestingly, all the seven quantified constituents of WSE were non-inhibitors of P-glycoprotein. The carcinogenic and mutagenic potential has a direct or indirect correlation with the molecular properties of the compounds. All constituents were found to be non-AMES toxic, which was also supported by our *in vitro* AMES (mutagenicity) assay of WSE. Further, these constituents were found to be non-carcinogenic, while acute oral toxicity prediction revealed that, except for withanoside IV and withanoside V, other constituents come under toxicity Class I. Here, the predicted carcinogenicity, acute rat toxicity, fish toxicity, and tetrahymena pyriformis toxicity are summarized in Table 2.

### 2.2. Quantification of Withania somnifera Root Extract

The quantitative estimation of WSE (*n* = 3) constituents, namely withanoside IV, withanoside V, withaferin A, 12-Deoxy-withastramonolide, withanolide A, withanolide B, and withanone, are depicted in Table 3.

### 2.3. Chromosomal Aberration (Genotoxicity) Assay of Withania somnifera Root Extract

*Withania somnifera* extract was evaluated at three different concentrations (0.25 mg/mL, 0.50 mg/mL, and 1.00 mg/mL) for chromosomal aberration assay as per the OECD 473 guideline. In the case of the untreated control, % chromosome aberration was found to be 4% without a metabolic activation system (Phase I) and with a metabolic activation system (Phase II). In the case of the negative control, it was found that % chromosome aberration in Phase I and Phase II activation system was 5%. In contrast, in the positive control, it was found that the % chromosome aberration in Phase I and Phase II activation system were 21% and 52%, respectively. Treatment with 0.25, 0.50, and 1.00 mg/mL WSE revealed that % chromosome aberration without the metabolic activation system was 5% while it was also found to be 5% with the metabolic activation system. The microscopical observations and data calculations demonstrate that the WSE did not cause chromosomal aberration more than the positive controls mitomycin C (Phase I) and cyclophosphamide (Phase II).

Further, it was found that the ratio between positive to negative control is more than two, while the WSE to the negative control is less than two. Hence, it was observed that WSE is non-cytogenetic in Phase I and Phase II studies. The results of chromosomal aberration are represented in Table 4.

### 2.4. Salmonella typhimurium Reverse Mutation Assay (AMES) Assay 

A mutagenicity study was performed to determine the potential of WSE to induce gene mutations compared to the negative control according to the plate incorporation test (Trial I) and pre-incubation test (Trial II). Results revealed that, when treated with WSE, no significant increase in colony numbers was observed in *Salmonella typhimurium* strains at any dose level, neither in the presence nor in the absence of metabolic activation. There was no tendency of higher mutation with increasing concentration of WSE. 

### 2.5. UHPLC-MS/MS Method Optimization 

The chromatographic method was optimized with the separation of seven analytes, including two internal standards, within a 14 min run. In the ESI-MS spectra analysis, the charge distributions can be altered by adding buffers. Adduct formation could occur depending on the Lewis basicity of the compound in the presence of NH4^+^, Na^+^, and K^+^ or if Li^+^ or Ag^+^ are intentionally added to the sample. Therefore, withanoside IV was observed at Rt 1.25 min with the molecular formula C_40_H_62_O_15_ having a molecular weight of 782.92 g/mol gives the ammonium ion adduct at *m*/*z* 800.45 [M+NH_4_]^+^, while withanoside V (C_40_H_62_O_14_) was observed at the Rt 2.86 min with the molecular weight of 766.92 g/mol that gives the ammonium ion adduct at *m*/*z* 784.45 [M+NH_4_]^+^. Further, five withanolides were observed at different retention times, such as withaferin A and 12-Deoxy-withastramonolide having a similar molecular formula C_28_H_38_O_6_ with a molecular weight of 470.61 g/mol, and both give [M+H]^+^ ion at *m*/*z* 471.25. Later, withanolide A (C_28_H_38_O_6_) was observed at Rt 6.02 min with a molecular weight of 470.61 g/mol that gives the ammonium ion adduct at *m*/*z* 488.30 [M+NH_4_]^+^, while withanone gives the prominent fragment at *m*/*z* 417.25. Withanolide B was observed at Rt 8.23 min, which has a molecular formula of C_28_H_38_O_5_ that gives ammonium ion adduct at *m*/*z* 472.30. Internal standards fluoxymesterone demonstrated the molecular ion peaks at *m*/*z* 337.20 gives the protonated ion [M+H]^+^ and difenoconazole was observed at *m*/*z* 406.10 that gives [M]^+^. Further, MS/MS parameters were optimized on ESI with precursor and product ions in the MRM mode analysis. The MS/MS parameters were optimized for each analyte, as a source temperature, collision energy, and gas flow, based on detected ions and mass parameters. All seven analytes, including internal standards, demonstrated an optimum response in positive ESI mode with good sensitivity, reproducibility, and fragmentation. The optimized mass transition ion pairs (*m*/*z*) for quantification were optimized as 800.45/459.30 for withanoside IV, 784.45/443.30 for withanoside V, 471.25/67.05 for withaferin A, 471.25/67.05 for 12-Deoxy-withastramonolide, 488.30/471.25 for withanolide A, 472.30/109.15 for withanolide B and 417.25/263.15 for withanone, 337.20/91.15 for IS1 (fluoxymesterone), and 406.10/336.90 for IS2 (difenoconazole) (Table 5, Figure S1).

### 2.6. Sample Pre-Treatment for Bioanalysis

Due to the complex nature of plasma, a sample pre-treatment procedure is needed before LC-MS/MS analysis to eliminate protein and interferences. In the present study, good resolution and higher recovery of analytes were achieved by analyzing the samples spiked with different biological matrices and extraction methods (liquid-liquid extraction, solid-phase extraction, and protein precipitation). The solid-phase extraction technique using clean-up was finalized for the quantification of analytes. 

### 2.7. UHPLC-MS/MS Method Validation

#### 2.7.1. Specificity

The specificity was determined by comparing the retention times in chromatograms of plasma samples (with or without analytes) after oral administration of WSE. No interference of endogenous substances was observed between the retention times of withanoside IV, withanoside V, withaferin A, 12-Deoxy-withastramonolide, withanolide A, withanolide B, withanone, IS1, and IS2, indicating reasonable specificity of the method. In this method, no carry-over effect was observed.

#### 2.7.2. Linearity and Lower Limit of Quantification (LLOQ)

The linearity of seven analytes with correlation coefficient (R^2^) was plotted, ranging from 0.9917 to 0.9976. The LLOQ of seven analytes was 3 ng/mL. The back-calculated concentration was within ±20% of nominal concentration, indicating the sensitivity of the proposed method. The calibration curve, LLOQ, and linearity for seven analytes are presented in Table S1.

#### 2.7.3. Precision and Accuracy

The intra-day and inter-day precision (% RSD) and accuracy (% RE) of the seven analytes were found within acceptability criteria (within 15%), demonstrating that the developed method was accurate, precise, and reproducible for all seven analytes. The results of accuracy and precision are presented in Table S2.

#### 2.7.4. Extraction Recovery and Matrix Effect

The percentage recovery of all analytes ranges from 92.06% to 99.96% (Table S3). Quality control samples were prepared at low (LQC), medium (MQC), and high (HQC) (*n* = 6) at three concentration levels (10, 75, 200 ng/mL). Matrix effect was found under the range of 85.28% to 109.77% (Table S4). 

#### 2.7.5. Dilution Integrity

Six replicates of samples at 25 μg/mL were diluted twenty times with blank rat plasma and were analyzed. The resulting concentrations were multiplied by the dilution factor of six. A negligible effect of dilution was observed (Table S5).

#### 2.7.6. Stability

Autosampler stability (for 36 h), freeze/thaw cycles (−80 °C to −25 °C), long-term (−80 °C for 60 days), bench-top stability (for 4 h), and processed stability (for 4 h) of analytes in plasma determined % RE and % RSD and were found within ±15%. Results suggest that all the analytes were stable under the prescribed storage conditions. Extraction recovery data are represented in Table S6.

### 2.8. In Vivo Pharmacokinetic Study

**Figure 2:**
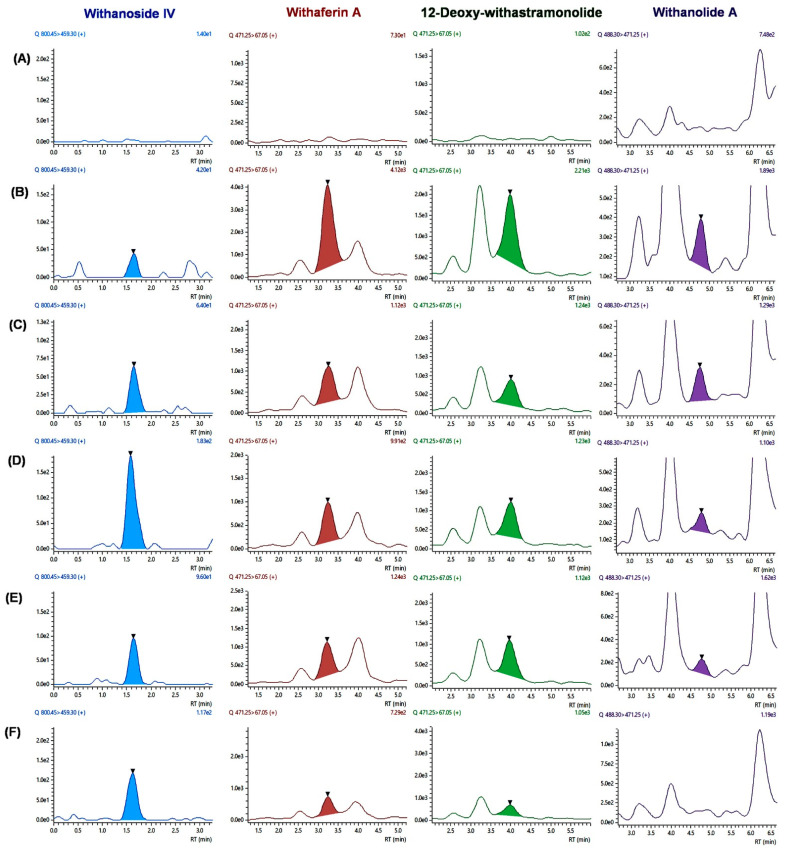
LC-MS/MS chromatogram of four constituents after oral administration of WSE at the dose of 500 mg/kg in rats; (**A**) blank plasma; chromatograms of individual constituents at different time points; (**B**) 15 min; (**C**) 30 min; (**D**) 45 min; (**E**) 1 h; and (**F**) 2 h.

**Figure 3:**
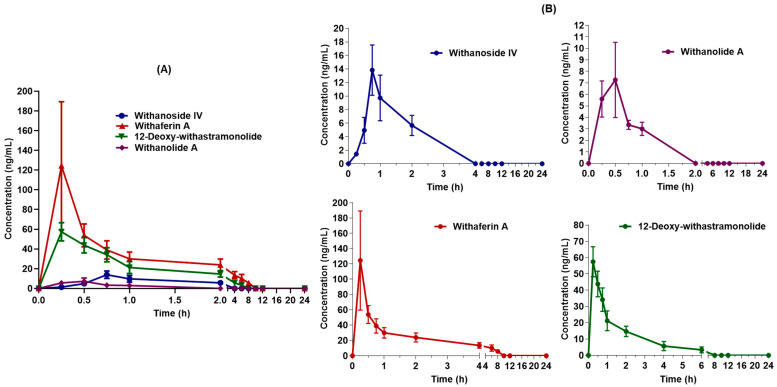
Mean plasma concentration vs. time curves of four constituents quantified (**A**) overlay; (**B**) individual constituents after oral administration of *Withania somnifera* extract at the dose of 500 mg/kg in male *Sprague Dawley* rats (Mean ± SD, *n* = 6).

The validated UHPLC-MS/MS method was applied to estimate seven constituents simultaneously after single-dose oral administration of WSE (500 mg/kg) (*n* = 6) in male *Sprague Dawley* rats. The four constituents were quantified at a first-time point (i.e., 15 min), excluding withanoside V, withanolide B, and withanone. LC-MS/MS chromatogram of four constituents after oral administration of WSE at the dose of 500 mg/kg in rats is shown in Figure 2. The mean plasma concentration vs. time curves of four constituents are shown in Figure 3. The pharmacokinetic parameters for the constituents of WSE were calculated and are listed in Table 6. The peak plasma concentrations (C*_max_*) were found to be 13.833 ± 3.727 ng/mL, 124.415 ± 64.932 ng/mL, 57.536 ± 7.523 ng/mL, and 7.283 ± 3.341 ng/mL for withanoside IV, withaferin A, 12-Deoxy-withastramonolide, and withanolide A, respectively, with observed T*_max_* of 0.750 ± 0.000, 0.250 ± 0.000, 0.291 ± 0.102, and 0.333 ± 0.129 h, respectively. Results indicate that withanosides and withanolides were rapidly absorbed from the stomach. The LLOQ values of withaferin A and 12-Deoxy-withastramonolide were found to be less than the C*_max_*/20 ratio, which indicates that the developed method was sensitive to determining the concentration of these constituents in the plasma. The area under the plasma concentration vs. time curve from 0–24 h (AUC_(0–24h)_) was found to be 13.960 ± 3.703, 161.180 ± 18.863, 82.866 ± 7.820, and 4.179 ± 1.032 h.ng/mL for withanoside IV, withaferin A, 12-Deoxy-withstramonolide, and withanolide A, respectively. The ratio of AUC_(0–24h)_ to AUC_(0–∞)_ was found to be 0.859 ± 0.057 and 0.904 ± 0.059 for withaferin A and 12-Deoxy-withastramonolide at nearly 1.00, which suggests that the sampling interval and calculated terminal half-life were appropriately selected. Further, the half-life (t_1/2_) of withanoside IV, withaferin A, 12-Deoxy-withstramonolide, and withanolide A was found to be 1.101 ± 0.272, 3.148 ± 0.612, 1.734 ± 0.505, and 0.728 ± 0.423 h and oral clearance (*Cl*/*F*) 0.176 ± 0.037, 0.026 ± 0.003, 0.016 ± 0.002, and 0.356 ± 0.090 (mg/kg)/(ng/mL)/h, respectively. Here, withanoside IV, withaferin A, and 12-Deoxy-withastramonolide demonstrated the greater extent of oral absorption from the stomach lining with a higher C*_max_*, lower half-life, and clearance. Withanoside IV has a higher molecular weight > 700 g/mol, yet polar due to the glucose moieties attached at the C3 position. It is known that the hydrophobic interior surface of the cell membrane (lipid bilayer) serves as a barrier to the passage of polar and high molecular weight molecules. Hence, withanoside IV was readily absorbed by passive diffusion, as it belongs to the biopharmaceutical classification system (BCS) Class-I (compounds with high solubility and high permeability). Further, the content of withanoside IV was 3.8715 mg/500 mg WSE, which is almost similar to withaferin A; however, a lower concentration in plasma was detected. This might be due to the gut glucosidase enzyme, which could hydrolyze compounds and form the aglycone part of withanoside IV with the loss of glycosidic linkages. Withaferin A and 12-Deoxy-withastramonolide were detected in the plasma up to 10 h, indicating that these constituents might have a lower elimination rate from the body. In contrast, the remaining two constituents, namely withanoside IV and withanolide A were not detected after 2 h, suggesting a low oral bioavailability of these constituents, which may be due to the extensive first-pass metabolism and rapid excretion from the body. Whereas withanoside V, withanolide B, and withanone were detected in the plasma after oral administration of the extract, the concentration of these constituents was found below LLOQ. 

### 2.9. Ex Vivo Permeability Study

#### Estimation of Permeability

A linear absorption pattern was observed for all seven markers in the initial lag phase and later showed a plateau. The dQ/dt was determined from the slope of the linear phase. Therefore, the apparent permeability coefficient (P*_app_*) was found to be 1.4174 × 10^−7^, 3.4254 × 10^−8^, 1.1252 × 10^−7^, 1.2221 × 10^−7^, 6.6487 × 10^−8^, 1.3065 × 10^−8^, 3.1746 × 10^−8^ cm/s for withanoside IV, withanoside V, withaferin A, 12-Deoxy-withastramonolide, withanolide A, withanolide B, and withanone, respectively (Table 7).

**Figure 4:**
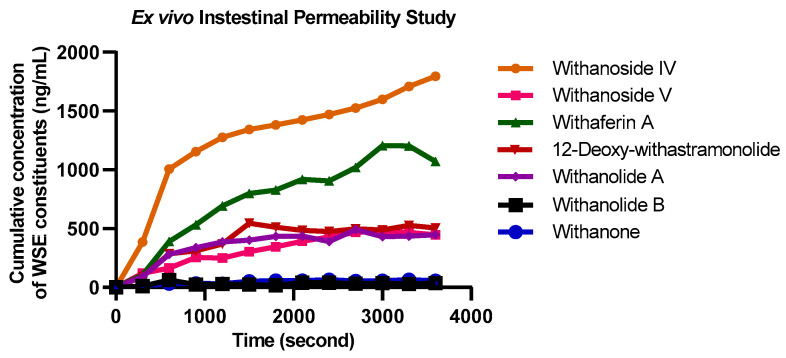
Overlay absorption kinetics of *Withania somnifera* extract constituents using everted intestine apparatus (Mean ± SD, *n* = 6).

In the present study, a simpler *ex vivo* permeability model was used to study the absorption pattern and apparent permeability characteristic using the ileocecal segment of the rat intestine. Considering the site-specific permeation of the xenobiotic agents with the pre-determined absorption window, a portable apparatus was developed to study permeability characteristics, and the length of the intestinal segment was kept at 3 cm. The study discloses the transport mechanism of seven prominent biomarkers of WSE across the intestinal barrier to illustrate drug absorption patterns. With advanced techniques, such as LC-MS/MS, these constituents were quantified upon permeation through the rat intestinal segment. The apparatus was attached to a 1 L reservoir and aeration pump to mimic the peristaltic movement and sink conditions. From the results, the constituents followed the passive diffusion absorption process. Initially, the constituents were readily absorbed and later achieved the steady-state concentration. Withanoside IV, withaferin A, and 12-Deoxy-withastramonolide have demonstrated maximum absorption from the donor compartment to the acceptor compartment. Here, with higher solubility, withanoside IV (Log *p* = 1.22) was readily transported across the intestinal barrier. At the same time, withanone and withanolide B were found at lower concentrations (Figure 4).

## 3. Discussion

*Withania somnifera* is a medicinal plant and is recognized as an ethnopharmacological functional food with diverse pharmacological properties. In pre-clinical studies, *Withania somnifera* root extract and its chemical constituents have proven to be active against several biological activities, such as aphrodisiac, adaptogenic, immunomodulatory, anti-inflammatory, anti-diabetic, hepatoprotective, anti-stress, anti-rheumatic, and neuroprotective [19,20,21,22,23,24,25,26]. Meanwhile, in clinical studies, WSE has been studied for insomnia, anxiety, stress, weight management, thyroid gland function, cardio-respiratory endurance, muscle strength, male and female sexual function recovery, bipolar disorders, and anti-aging activity [27,28,29,30]. This dietary supplement has been regulated and requires validated ADMET data to understand the bioavailability of WSE constituents. The pharmacokinetic parameters of WSE are still unexplored collectively with their significant constituents in the living system. The major phytoconstituents in ‘Ashwagandha’ root extract are withanoside IV, withanoside V, withaferin A, 12-Deoxy-withastramonolide withanolide A, withanolide B, and withanone [31]. Based on that, a precise, sensitive, and accurate bioanalytical method was developed to estimate seven withanosides and withanolides simultaneously in rat plasma. The validation of this novel bioanalytical method was carried out according to the USFDA/EMA guidelines in the presence of two internal standards, which results in linearity with R^2^ > 0.990. The lower limit of quantification (LLOQ) was found to be 3 ng/mL for all seven constituents of WSE. 

Upon oral administration of WSE, except withanoside V, withanolide B, and withanone, four constituents were quantified appropriately *in vivo*, enabling us to understand how the body reacts to these constituents individually. Recently, Dai and Tianming et al. (2019) studied the oral bioavailability and first-pass metabolism of withaferin A (10 mg/kg oral dose; 5 mg/kg I.V. dose) in rats using LC-MS/MS and Q-TRAP. Results revealed that upon oral administration of withaferin A in male rats, a half-life of 7.6 ± 3.3 h and bioavailability of 32.4 ± 4.8% was observed. The C*_max_* and T*_max_* were found to be 619 ± 125 ng/mL and 0.11 ± 0.07 h [32]. Thaiparambil and Jose T. et al. (2011) reported a pharmacokinetic study of withaferin A on *Balb*/*C* mice at a dose of 4 mg/kg followed by a single intraperitoneal (I.P.) route of administration. The C*_max_* was found to be 1.8 µM at T*_max_* (0.083 h). The plasma half-life of withaferin A was 1.36 h, and the systemic exposure, AUC_(0–t)_, was 1.09 µM.h. Clearance from plasma was rapid (0.151 L/h). Withaferin A was undetectable at 24 h and was found below LLOQ in all mice up to 6 h [33]. Gambhir L. et al. (2015) reported a pharmacokinetics and biodistribution study of withaferin A upon intraperitoneal administration using HPLC, which lacks the *in vivo* pharmacokinetics estimation of all seven biomarkers from WSE simultaneously [34]. Patil, Dada et al. (2013) developed a bioanalytical method to determine only withaferin A and withanolide A upon oral administration of the *Withania somnifera* aqueous extract at a dose of 1000 mg/kg (equivalent to 0.4585 mg/kg of withaferin A and 0.4785 mg/kg of withanolide A) in mice using high-performance-liquid chromatography-tandem mass spectrometry. These results suggest that the time required to reach maximum concentration (T*_max_*), and plasma half-life (t_1/2_) of withaferin A was found to be 20.00 min and 59.92 ± 15.90 min, respectively, while for withanolide A, T*_max,_* and t_1/2_ were found to be 10.00 min and 45.22 ± 9.95 min, respectively [35]. Therefore, in our study, the time required to reach a maximum plasma concentration (C*_max_*) and plasma half-life (t_1/2_) of withaferin A were found to be 0.250 ± 0.000 h and 3.148 ± 0.612 h, respectively, while T*_max_* and t_1/2_ for withanolide A were found to be 0.333 ± 0.129 h and 0.728 ± 0.423 h. The previous bioanalytical methods were developed specifically for withaferin A and withanolide A, and pharmacokinetics are more limited to withaferin A, whereas, in our study, a validated bioanalytical method was developed to determine seven major phytoconstituents simultaneously from a commercial *Withania somnifera* root extract. This validated bioanalytical method was later applied to determine the pharmacokinetic parameters of WSE chemical constituents, namely withanoside IV, withaferin A, 12-Deoxy-withastramonolide, and withanolide A in rat plasma. 

At a given optimum dose (500 mg/kg), a genotoxicity and mutagenicity study of WSE was carried out. Additionally, to study the permeation characteristics of these constituents across the intestinal barriers, an *ex vivo* assay using excised everted rat intestinal segment was developed, expressing the advantages over most of the influx and efflux transporters, similar to an *in vivo* study. Seven markers of WSE were found capable of permeating the intestinal mucosa, and steady-state concentration was achieved after a lag time. At the same time, the amount of withanone and withanolide B were detected in lower concentrations compared to other constituents. Consequently, none of the constituents were found to be carcinogenic, mutagenic, or hepatotoxic in the ADMET study. Our previous study reported acute and sub-acute oral toxicity studies using female *Wistar* rats by orally administering WSE, indicating that the LD_50_ value of WSE was > 2000 mg/kg with no toxic effects on the organs of animals [36]. Hence, WSE did not exhibit any mutagenicity or genotoxicity, as per the *in vitro* assay. 

The presence of glucose moiety at the C3 atom, with a high-water solubility and rapid elimination of withanoside IV from the systemic circulation, could be a potential reason for lower toxicity supported by the *in vivo* pharmacokinetic data, in which withanoside IV was eliminated at 2 h. Similarly, it was observed that all the constituents were rapidly absorbed from the gastrointestinal tract. The higher molecular weight (>450 g/mol) makes these compounds prone to passive diffusion from the stomach and intestine, supported by the *ex vivo* model. The time-dependent and steady-state absorption of constituents was observed from the intestinal lumen. Overall, results suggest a strong correlation between *in silico* ADMET, *in vivo* pharmacokinetics, and the *ex vivo* permeability study. The collective results of the studies suggest that this validated bioanalytical method could be helpful to explore a clinical pharmacokinetic study of WSE supplements in humans.

## 4. Materials and Methods

### 4.1. Chemicals and Reagents

Withanoside IV (purity > 93.00%) and withanolide A (purity > 99.00%) were purchased from USP, Rockville, MD, USA; withanoside V (purity > 97.60%), withanolide B (purity > 95.00%), 12-Deoxy-withastramonolide (purity > 95.90%), and withanone (purity > 93.90%) were procured from Natural Remedies Pvt. Ltd., Bengaluru, India. Withaferin A (purity > 97.60%) was procured from Chromadex, Los Angeles, CA, USA. Fluoxymesterone (FMC, internal standard, IS1) with the purity > 98.82% and difenoconazole (DFC, internal standard, IS2) with the purity > 95.90% were purchased from Clearsynth, Mumbai, Maharashtra, India, and Sigma-Aldrich, St. Louis, MO, USA, respectively. Acetonitrile, methanol, and water (MS grade) were purchased from JT Baker, Radnor, PA, USA. Formic acid was purchased from Honeywell, Charlotte, NC, USA.

### 4.2. In Silico Absorption, Distribution, Metabolism, Excretion and Toxicity (ADMET), and Molecular Properties Prediction 

Poor pharmacokinetics and adverse drug events are directly or indirectly related to the poor prognosis of drug/chemical constituents. Therefore, an *in silico* ADMET prediction was performed to individually estimate the pharmacokinetics/toxicity of seven constituents of WSE. AdmetSAR is an accessible online server that predicts ADMET properties, such as human intestinal absorption (HIA), blood-brain barrier (BBB+) penetration, Caco-2 permeability, biodegradation, AMES toxicity, carcinogenicity, rat acute oral toxicity, and so on. The ADMET properties of WSE constituents were estimated using the admetSAR online database (http://lmmd.ecust.edu.cn/admetsar2/, accessed on 14 January 2022) to predict ADMET properties. It provides inclusive data for different entities linked to known ADMET properties. Molecular property prediction and Lipinski’s rule of five (ROF) were used to evaluate drug-likeness and/or to determine whether a chemical constituent with a particular pharmacological activity would make it an orally active drug for humans. Molinspiration software (www.molinspiration.com, accessed on 14 January 2022) was used to determine fundamental and molecular properties of these constituents, such as the hydrogen bond donor/acceptor, molecular weight, lipophilicity, etc. [37,38,39].

### 4.3. Withania somnifera Extract Preparation and Quantification

*Withania somnifera* roots were procured from Neemuch (MP), India. A voucher specimen was deposited at the Botanical Survey of India (Jodhpur, India) and was authenticated (BSI/AZRC/ I.12012/Tech/19-20/PI. Id/671). The root extract and quantitative assay using HPLC-PDA were performed as per our reported literature [40]. 

### 4.4. In Vitro Toxicity Studies

#### 4.4.1. Chromosomal Aberration Assay of *Withania somnifera* Root Extract

The chromosomal aberration assay was performed to evaluate WSE for the induction of structural deformities in the chromosomes, such as breaking and exchange. For that, Phase I and Phase II genotoxicity study was performed at the three concentrations (0.25, 0.50, and 1.00 mg/mL) in the presence and absence of the metabolic activation system along with the negative and positive control, as per the OECD 473 guideline. After completing the study, chromosomal slides were observed under 100×, and the % aberrated chromosomes were calculated. Further, the mitotic index (MI), relative cell growth (RCG), relative mitotic index (RMI), and % aberration were also calculated upon treatment with WSE [41]. In without metabolic activation system (Phase I), mitomycin C, and with metabolic activation system (Phase II), cyclophosphamide was used as a positive control. 

#### 4.4.2. AMES Toxicity Study of *Withania somnifera* Root Extract

This study was performed to investigate the potential of WSE to induce gene mutations compared to the negative control according to the plate incorporation test (Trial 1) and the pre-incubation test (Trial II) using the *Salmonella typhimurium* strains TA 1537, TA 1535, TA 98, TA 100, and TA 102. The assay was performed in two independent experiments with or without a liver microsomal activation system. Each concentration, including the negative and positive control, was tested in triplicate. Based on the solubility and precipitation test, the results for eight different concentrations, namely, 0.002, 0.005, 0.016, 0.050, 0.158, 0.501, 1.582, and 5.000 mg/plate were selected for pre-experiments. Later, the following concentrations, 0.016, 0.051, 0.158, 0.501 and 1.582 mg/plate, were selected for the presence of metabolic activation (+S9) and in the absence of metabolic activation (-S9) [42].

### 4.5. Development and Validation of UHPLC-MS/MS Method 

#### 4.5.1. Instrumental and Chromatographic Conditions

The LC-MS/MS analysis was carried out using the Shimadzu Nexera X2 UHPLC system (Shimadzu Tech., Kyoto, Japan), which consists of a degasser DGU-20A5R, quaternary pump LC-30AD, autosampler SIL-30AC, and column oven CTO-20AC with a diode-array detector (SPD-M20A), coupled with LCMS-8045 (Shimadzu Tech., Kyoto, Japan), and triple-quadrupole mass detector equipped with a thermally assisted ESI source. An outlet of the PDA detector was connected with a splitter, which split the flow in the ratio of 5:1 mL/min The separation of individual constituents was obtained from Dr. Maisch GmbH; ReproSil Gold 100C18-XBD, 50 × 4.6 mm; 1.8 µm and associated with a Phenomenex guard column (temperature was maintained at 28 °C) within 14 min run time. The mobile phase composition was 0.1% formic acid in water (A) and acetonitrile (B) with a flow rate of 0.5 mL/min, and the injection volume was kept at 2 µL. The gradient elution procedure was set as follows: 0.00 min, 40% B; 0.00–2.40 min, 45% B; 2.40–4.46 min, 50% B; 4.46–7.00 min, 100% B; 7.00–9.00 min, 100% B; 9.00–11.00 min, 40% B; 11.00–14.00 min, 40% B. 

Mass analysis (LC-MS/MS) was carried out in scan mode, and the MS/MS analyses in both positive and negative ion modes (Table 5). The interface for ESI was set to 300 °C. The desolvation line (DL) and heat block temperature were set to 250 °C and 300 °C, respectively. Nebulizing gas flow, 3.00 L/min; heating gas flow, 10.00 L/min; drying gas flow, 10.00 L/min The operation and data generation have been carried out using Lab Solution and Lab Solution Insight software version 3.2 (Shimadzu Corporation, Kyoto, Japan).

#### 4.5.2. Preparation of Stock and Working Standards

The seven individual stock solutions (0.1 mg/mL) of withanoside IV, withanoside V, withaferin A, 12-Deoxy-withastramonolide, withanolide A, withanolide B, and withanone with an individual stock solution of IS1 fluoxymesterone (1.0 mg/mL) and IS2 difenoconazole (0.01 mg/mL) were prepared using LC-MS grade methanol for the preparation of the calibration curve (CC) for both standard and quality control (QC) samples. The primary stock dilutions and working solutions were prepared using methanol ranging from 3.00 to 400.00 ng/mL. Further, IS1 (0.005 mg/mL) and IS2 (0.0001 mg/mL) stock solutions were prepared following the similar method. 

#### 4.5.3. Preparation of Calibration Standards and Quality Control Samples

The calibration standards were prepared by spiking 400 µL plasma in methanol and vortexed for 2 min The samples were centrifuged at 5000 rpm for 10 min An aliquot of 85 µL of supernatant was spiked with 0.005 mg/mL of fluoxymesterone (IS1), 0.0001 mg/mL of difenoconazole (IS2), and 5 µL of working solution, respectively. A ten-point calibration curve (3, 5, 10, 25, 50, 75, 100, 200, 300, and 400 ng/mL) was constructed by plotting the ratio of the peak areas of withanoside IV, withanoside V, withaferin A, 12-Deoxy-withastramonolide, withanolide A, withanolide B, and withanone against the nominal concentrations of the calibration standards in the control matrix. The data were subjected to linear regression with a 1/X² weighing.

#### 4.5.4. Sample Pre-Treatment for UHPLC-MS/MS Analysis

The solid-phase extraction (SPE) method was utilized to extract withanoside IV, withanoside V, withaferin A, 12-Deoxy-withastramonolide, withanolide A, withanolide B, and withanone. An aliquot of 85 μL plasma was spiked with 5 μL mix standards, 5 μL of IS1 and IS2. Methanol (400 μL) was added to this mixture, vortexed for 1–2 min, and centrifuged at 5000 rpm for 10 min After centrifugation, 300 µL supernatant was collected into 1.5 mL Eppendorf tubes, then 300 µL methanol was added and vortexed for 1–2 min Bond Elute C18 SPE cartridges were conditioned with 1 mL methanol passed through the SPE manifold, followed by sample loading into the cartridges. They were further washed twice with methanol and passed through the SPE manifold, loading the sample into cartridges of 0.1% formic acid solution (1 mL) to activate the targeted analyte. After that, the reconstitution of pre-treated samples was achieved using methanol (300 µL). The final solution was collected into HPLC vials, and the 2 µL solution was injected into the LC-MS/MS system for bio-analysis.

#### 4.5.5. UHPLC-MS/MS Method Validation

The validation for specificity, linearity, extraction recovery, matrix effects, precision, accuracy, dilution effect, and stability of the developed bioanalytical method was performed as per the US Food and Drug Administration (2018) and European Medicine Agency (2011) guidelines [43,44].

#### 4.5.6. Selectivity, Linearity, and Lower Limit of Quantification (LLOQ)

The selectivity was assessed by analyzing endogenous components in the elution zone of analytes and internal standard (IS) from the chromatogram. Rat plasma samples (*n* = 6) were spiked at the LLOQ levels. After oral dosing, the samples collected from animals were processed as per the procedure mentioned above, followed by solid-phase extraction (SPE) and LC-MS/MS method. Thereafter, a ten-points calibration curve for the individual standard was prepared and plotted to the IS response to individual constituents against the nominal concentration of all constituents by applying partial least-square linear regression analysis. The lower limit of quantification (LLOQ) was calculated from the above calibration plot by calculating the accuracy and precision.

#### 4.5.7. Matrix Effect, Dilution Integrity, and Extraction Recovery

The matrix effect was evaluated by comparing the MS/MS signals for individual components and internal standards with spiked blank plasma at three different quality control concentration levels of standards. Subsequently, the carry-over effect was determined to ensure the absence of an analytical signal between two consecutive samples in a sequence due to the traces of analyte from previous runs. Dilution integrity was evaluated by injecting 500 ng/mL of each analyte against blank plasma, followed by calculating % relative error (% RE) and % relative standard deviation (% RSD). The comparison of unextracted plasma samples determined the extraction recovery at three concentration levels, namely, LQC, MQC, and HQC, against the extracted samples to evaluate the extraction recovery of the chemical constituents of WSE. 

#### 4.5.8. Accuracy, Precision, and Stability 

Six replicates of quality control samples were analyzed at four concentration levels (LLOQ, LQC, MQC, and HQC; *n* = 6) on the same day (intra-day) and repeated for three consecutive days (inter-day) (Table S2). The precision was expressed as the relative standard deviation (% RSD), and accuracy was reported as the relative error (% RE). Later, these samples were analyzed for stability testing under the following conditions: three complete freeze/thaw cycles (−80 °C to 25 °C), long-term sample storage (−80 °C for 60 days), processed sample, and bench-top (25 °C for 4 h). At the same time, autosampler stability was evaluated at 4 °C for 48 h in the autosampler rack.

### 4.6. In Vivo Pharmacokinetic Study 

The pharmacokinetic study was carried out on male *Sprague Dawley* rats (weighing 200–250 gm) at the Crystal Biological Solution, Pune, Maharashtra, India (Animal House Registration no. 2030/P.O./RcBiBt/S/18/CPCSEA). Animals were quarantined for two weeks in standard conditions 24 ± 1 °C, relative humidity of 56–66%, and 12 h dark and light cycles. Food pellets and tap water were provided *ad libitum* throughout the experimentation. The protocol to carry out *in vivo* pharmacokinetics was approved by The Institutional Animal Ethics Committee (IAEC) of Crystal Biological Solution, Pune, Maharashtra, India. (Approval No. CRY/2021/015). Eighteen animals were subdivided into three groups, (*n* = 6) animals in each group. *Withania somnifera* extract suspension was prepared in water for oral administration, and the dose was calculated based on the acute oral toxicity study (500 mg/kg; single dose). After dosing, animals were anesthetized using Ketamine (75 mg/kg) + Xylazine (10 mg/kg) by the intraperitoneal route, and the blood samples (0.1 mL) were collected from each group, followed by the retro-orbital venous plexus method (ROP) at different time points (i.e., 0 min, 15 min, 30 min, 45 min, 1 h, 2 h, 4 h, 6 h, 8 h, 10 h, 12 h, and 24 h) using Heparin tubes and further centrifuged at 10,000 rpm for 15 min at 4 °C to collect the plasma samples. The separated plasma samples were transferred into Eppendorf tubes and stored at −80 °C in a deep freezer until analysis. Ten pharmacokinetic parameters were determined using PK solver 2.0, USA [45,46,47]. The maximum observed concentration (C*_max_*) and maximum observed time (T*_max_*) were calculated directly from the experimental results. The elimination rate constant (K*_el_*) was calculated from the slope of the linear regression of Log concentrations as a function of time. The half-life (t_1/2_) was calculated as 0.693/K*_el_*. The area under the curve (AUC_(0–t)_) was calculated from the linear curve following the trapezoidal rule, while AUC_(0–∞)_ was calculated by adding C*_t_*/λ, with C*_t_* being the final measurable concentration. Oral clearance (*Cl*/*F*) was obtained from the equation *Cl*/*F* = dose/AUC_(0–∞)_. Mean residence time (MRT) was calculated as AUMC_(0–∞)_/AUC_(0–∞)_, where AUMC_(0–∞)_ is the area under the first moment curve. Each plasma sample was analyzed in triplicate, and the results are presented as a mean ± standard deviation (SD). The pharmacokinetic parameters as the average of six rats ± SD. The mean plasma concentration Vs. time curve (mean ± SD) was prepared using Prism 8.2, (GraphPad, San Diego, CA, USA).

### 4.7. Ex Vivo Permeability Study

#### 4.7.1. Everted Rat Intestine Apparatus

The glass apparatus for permeability study was developed as per the previously reported procedure with slight modifications [48]. The everted intestinal segment was mounted on the apparatus and setting it in the beaker. The internal surface of the glass tubes serves as the mucosal compartment, and the beaker serves as the serosal compartment. Animals were humanly sacrificed by cervical dislocation followed by a midline incision. The abdominal cavity was opened carefully, and the intestine was maneuvered to identify the ileocecal junction. A 7 cm long intestinal segment distant to the ileocecal junction (5–6 cm) was excised by carefully removing the mesenteric attachments without causing any damage to the intestinal architecture. The segment was cleaned using the Kreb’s Ringer Bicarbonate (KRB) buffer medium and was stored in KRB buffer in a petri dish. The cleaned segment was then everted gently using a glass rod. Finally, a 3 cm long intestinal segment was used to perform the permeability study [49,50].

#### 4.7.2. Permeability Determination

The tissue was mounted on the apparatus and was placed in a 1000 mL beaker containing 2 mg/mL WSE suspension. The intestinal segment was perfused from the mucosal end using the KRB buffer. The apparatus (beaker with tissue) was placed on the magnetic stirrer followed by continuous stirring using a magnetic bead at 25 rpm to mimic the peristaltic movement of the stomach at 37 °C with appropriate aeration. The sample collection at different time points was carried out every 5 min interval for 1 h. The collected samples were further diluted and analyzed using LC-MS/MS. 

#### 4.7.3. Matrix Effect and Sample Preparation for *Ex Vivo* Permeability Study

The matrix effect was determined by spiking all analytes at LQC and HQC levels in the sample matrix and absence matrix, and it was found to be 85% to 115% for each analyte. The sample was diluted to 20-fold in ACN and injected 2 µL into LC-MS/MS. Samples were stored at −20 °C until analysis using validated UHPLC-MS/MS. The permeability was obtained by plotting the cumulative amount transported (ng) across the gut sac against time (second).

#### 4.7.4. Permeability Calculation

The calculation of apparent permeability (P*_app_*) was carried out using the following Equation (1):

P*_app_* = dQ/dt × 1/A × C_0_ (1)

where P*_app_*: apparent permeability co-efficient; dQ/dt: the cumulative amount of drug (Q) appearing in the acceptor (serosal) compartment as a function of time and was obtained from the slope of the linear portion of the amount transported-versus-time plot. A: Surface area of the intestine (cm^2^) (0.18 cm as radius) and C_0_: Initial concentration of drug in the donor compartment (ng/mL).

## 5. Conclusions

*Withania somnifera* extract (WSE) is a popular dietary supplement containing withanolides and withanosides. This nutritional supplement has been regulated globally and requires validated bioanalytical methodology and pharmacokinetic parameters. In the present study, for the first time, an accurate, sensitive, and validated bioanalytical method, as per the USFDA/EMA guidelines, was developed to simultaneously estimate seven constituents in rat plasma from WSE using UHPLC-MS/MS. The method demonstrated excellent linearity (R^2^ > 0.9917) with a lower limit of quantification (LLOQ) of 3 ng/mL for withanosides and withanolides in the presence of internal standards (fluoxymesterone and difenoconazole). The method was further applied to determine the pharmacokinetic parameters of withanolides and withanosides from WSE at the dose of 500 mg/kg. Genotoxicity data revealed that the ratio between positive and negative control is more than two, while the sample to negative control is less than two. Hence, it can be concluded that WSE is non-cytogenetic in Phase I and Phase II studies. Further, an *ex vivo* intestinal permeability suggests a time-dependent absorption of withanosides and withanolides through intestinal lumen, similar to BCS class I drugs with high solubility and permeability. Therefore, the report discloses the simultaneous estimation of two withanosides and five withanolides in a robust and accurate bioanalytical method. This is the first report where a validated UHPLC-MS/MS-based bioanalytical method demonstrated excellent specificity, linearity, precision, and accuracy. The pharmacokinetic and *ex vivo* permeability study results could be helpful for clinical trials of WSE in humans.

## Figures and Tables

**Table 1:** Lipinski’s rule of five for constituents of *Withania somnifera* standardized root extract.

Constituents	*Log *p*	*TPSA	**natoms	*M.W.	**nOH	**nOHNH	**nviolations	**nrotb	Volume
Withanoside IV	1.22	245.29	55	782.92	15	9	3	9	714.61
Withanoside V	2.46	225.06	54	766.92	14	8	3	8	706.35
Withaferin A	3.86	96.36	34	470.61	6	2	0	3	442.38
12-Deoxy-withastramonolide	3.86	96.36	34	470.61	6	2	0	3	442.38
Withanolide A	4.15	96.36	34	470.61	6	2	0	2	441.81
Withanolide B	5.10	76.13	33	454.61	5	1	1	2	434.12
Withanone	4.15	96.36	34	470.61	6	2	0	2	441.81

*Log P—partition coefficient; *TPSA—topological polar surface area; **natoms—number of atoms; *M.W.—molecular weight (g/mol); **nOH—hydrogen bond donor; **nOHNH—hydrogen bond acceptor; **nviolations—number of violations in Lipinski’s rule; **nrotb—number of rotational bonds.

**Table 2:** Results of ADMET prediction of *Withania somnifera* extract constituents.

ADMET Parameters	Withanoside IV		Withanoside V		Withaferin A		12-Deoxy-withastramonolide		Withanone		Withanolide A		Withanolide B	
	Results	Probability	Results	Probability	Results	Probability	Results	Probability	Results	Probability	Results	Probability	Results	Probability
Blood-brain barrier (BBB)	BBB	0.7718	BBB	0.7718	BBB+	0.8697	BBB+	0.8945	BBB+	0.8327	BBB+	0.8327	BBB+	0.9304
Human intestinal absorption (HIA)	HIA+	0.7051	HIA+	0.7051	HIA+	0.8086	HIA+	0.8393	HIA+	0.8951	HIA+	0.8951	HIA+	0.8990
Caco-2 permeability	Caco2-	0.9403	Caco2-	0.9403	Caco2-	0.6967	Caco2-	0.7345	Caco2-	0.7156	Caco2-	0.7156	Caco2-	0.5829
P-glycoprotein substrate	Substrate	0.8737	Substrate	0.8737	Substrate	0.7995	Substrate	0.8344	Substrate	0.8467	Substrate	0.8467	Substrate	0.7962
P-glycoprotein inhibitor	Non inhibitor	0.7603	Non inhibitor	0.7605	Non inhibitor	0.6149	Non inhibitor	0.7933	Non inhibitor	0.9071	Non inhibitor	0.9071	Non inhibitor	0.8898
Renal organic cation transporter	Non inhibitor	0.8153	Non inhibitor	0.8153	Non inhibitor	0.7575	Non inhibitor	0.7983	Non inhibitor	0.8620	Non inhibitor	0.8620	Non inhibitor	0.8440
Subcellular localization	Mitochondria	0.8076	Mitochondria	0.8076	Mitochondria	0.7714	Mitochondria	0.7267	Mitochondria	0.6830	Mitochondria	0.6830	Mitochondria	0.6784
*CYP450 2C9 substrate	Non substrate	0.8700	Non substrate	0.8000	Non substrate	0.8159	Non substrate	0.8166	Non substrate	0.8342	Non substrate	0.8342	Non substrate	0.7980
*CYP450 2D6 substrate	Non substrate	0.8905	Non substrate	0.8905	Non substate	0.8651	Non substate	0.8821	Non substate	0.8876	Non substate	0.8876	Non substate	0.8726
*CYP450 3A4 substrate	Substrate	0.7167	Substrate	0.7167	Substrate	0.7312	Substrate	0.7254	Substrate	0.7201	Substrate	0.7201	Substrate	0.7247
*CYP450 1A2 inhibitor	Non inhibitor	0.9243	Non inhibitor	0.9243	Non inhibitor	0.7829	Non inhibitor	0.8301	Non inhibitor	0.7538	Non inhibitor	0.7538	Non inhibitor	0.6899
*CYP450 2C9 inhibitor	Non inhibitor	0.9335	Non inhibitor	0.9335	Non inhibitor	0.8867	Non inhibitor	0.8752	Non inhibitor	0.8586	Non inhibitor	0.8586	Non inhibitor	0.9034
*CYP450 2D6 inhibitor	Non inhibitor	0.9467	Non inhibitor	0.9467	Non inhibitor	0.9517	Non inhibitor	0.9504	Non inhibitor	0.9560	Non inhibitor	0.9560	Non inhibitor	0.9541
*CYP450 2C19 inhibitor	Non inhibitor	0.9392	Non inhibitor	0.9392	Non inhibitor	0.9390	Non inhibitor	0.9390	Non inhibitor	0.8921	Non inhibitor	0.8921	Non inhibitor	0.9138
*CYP450 3A4 inhibitor	Non inhibitor	0.9495	Non inhibitor	0.9495	Non Inhibitor	0.8547	Non inhibitor	0.7286	Non inhibitor	0.7609	Non inhibitor	0.7609	Non inhibitor	0.7687
*CYP inhibitory promiscuity	Low CYP inhibitory promiscuity	0.9590	Low CYP inhibitory promiscuity	0.9590	Low CYP inhibitory promiscuity	0.9338	Low CYP inhibitory promiscuity	0.9564	Low CYP inhibitory promiscuity	0.9760	Low CYP inhibitory promiscuity	0.9760	Low CYP inhibitory promiscuity	0.9787
Human ether-a-go-go-related gene inhibition	Weak inhibitor	0.9442	Weak inhibitor	0.9442	Weak inhibitor	0.9703	Weak inhibitor	0.9751	Weak inhibitor	0.9855	Weak inhibitor	0.9855	Weak inhibitor	0.9796
AMES toxicity	Non-AMES toxic	0.9541	Non-AMES toxic	0.9541	Non-AMES toxic	0.6551	Non-AMES toxic	0.9195	Non-AMES Toxic	0.7270	Non-AMES Toxic	0.7270	Non-AMES Toxic	0.8562
Carcinogens	Non- carcinogens	0.9653	Non- carcinogens	0.9653	Non- carcinogens	0.9549	Non- carcinogens	0.9563	Non- carcinogens	0.9650	Non- carcinogens	0.9650	Non- carcinogens	0.9578
Fish toxicity	High FHMT	0.9600	High FHMT	0.9600	High FHMT	0.9426	High FHMT	0.9557	High FHMT	0.9778	High FHMT	0.9778	High FHMT	0.9773
Tetrahymena pyriformis toxicity	High TPT	0.9987	High TPT	0.9987	High TPT	0.9898	High TPT	0.9946	High TPT	0.9851	High TPT	0.9851	High TPT	0.9849
Honeybee toxicity	High HBT	0.8380	High HBT	0.8380	High HBT	0.7981	High HBT	0.7908	High HBT	0.7951	High HBT	0.7951	High HBT	0.8076
Biodegradation	Not readily biodegradable	0.9632	Not readily biodegradable	0.9632	Not readily biodegradable	0.9931	Not readily biodegradable	0.9923	Not readily biodegradable	0.9944	Not readily biodegradable	0.9944	Not readily biodegradable	0.9941
Acute oral toxicity	III	0.4565	III	0.4565	I	0.5780	I	0.6043	I	0.4368	I	0.4368	I	0.3632
Carcinogenicity (Three-class)	Non required	0.6109	Non required	0.6109	Non required	0.5377	Non required	0.5056	Non required	0.5461	Non required	0.5461	Non required	0.5543
Aqueous solubility (Log S)	−4.2128		−4.2128		−4.2028		−4.3532		−4.6120		−4.6120		−4.9110	
Caco-2 permeability (Log *p_app_*, cm/s)	−0.4407		−0.4407		0.7051		0.7306		0.8936		0.8936		1.1247	
Rat acute toxicity (LD_50_, mol/kg)	3.8118		3.8118		3.5404		3.4799		3.2351		3.2351		3.1455	
Fish toxicity (pLC_50_, mg/L)	1.1264		1.1264		0.7353		0.8683		0.8987		0.8987		0.6888	
Tetrahymena pyriformis toxicity (pIGC_50_, µg/L)	0.9363		0.9363		0.9439		0.9448		0.7361		0.7361		0.7863	

*CYP2C9-cytochrome P450 2C9; CYP2D6-cytochrome P450 2D6; CYP3A4-cytochrome P450 3A4; CYP1A2-cytochrome P450 1A2; CYP2C9-cytochrome P450 2C9; CYP2D6-cytochrome P450 2D6, CYP2C19-cytochrome P450 2C19; CYP3A4-cytochrome P450 3A4; CYP-cytochrome P450; CYP inhibitory promiscuity-cytochrome inhibitory promiscuity.

**Table 3:** Quantification of *Withania somnifera* extract (WSE) (*n* = 3; %, *w*/*w*).

Analytes	Content (%)
Withanoside IV	0.7743 ± 0.04
Withanoside V	0.9139 ± 0.03
Withaferin A	0.9682 ± 0.06
12-Deoxy-withastramonolide	0.3012 ± 0.02
Withanolide A	0.5102 ± 0.04
Withanolide B	0.1586 ± 0.04
Withanone	0.0042 ± 0.00

**Table 4:** Chromosomal aberration assay of *Withania somnifera* root extract.

**Without Metabolic Activation (Phase I)**					
Experimental condition	**RCG (%)	**MI	**RMI (%)	% Aberration	Ratio with Negative
Untreated control	88	62	112	4	0.80
Negative control	100	55	100	5	1.00
Positive control (Mitomycin C)	99	57	103	21	4.20
*WSE (0.25 mg/mL)	99	55	100	5	1.00
*WSE (0.50 mg/mL)	102	55	99	5	1.00
*WSE (1.00 mg/mL)	91	62	112	5	1.00
**With Metabolic Activation (Phase II)**					
Experimental condition	**RCG (%)	**MI	**RMI (%)	% Aberration	Ratio with Negative
Untreated control	107	57	94	4	0.80
Negative control	100	61	99	5	1.00
Positive control (Cyclophosphamide)	112	52	85	52	10.40
*WSE (0.25 mg/mL)	109	57	94	5	1.00
*WSE (0.50 mg/mL)	107	55	90	5	1.00
*WSE (1.00 mg/mL)	106	58	94	5	1.00

*WSE—*Withania somnifera* root extract; **RCG—relative cell growth; **MI—mitotic index; **RMI—relative mitotic index.

**Table 5:** Precursor/product ion pairs and parameters for multiple reaction monitoring (MRM) of WSE constituents (Figure S1).

Sr. No.	Analyte	Retention Time (Rt) Min.	Molecular Formula	Monoisotopic Mass	Precursor (*m*/*z*)	Product (*m*/*z*)	*Q1 Pre Bias (eV)	*CE (eV)	*Q3 Pre Bias (eV)
1	Withanoside IV	1.25	C_40_H_62_O_15_	782.40	800.45	459.30	−18.0	−23.0	−22.0
						621.35	−18.0	−16.0	−18.0
2	Withanoside V	2.87	C_40_H_62_O_14_	766.41	784.45	443.30	−22.0	−23.0	−22.0
						425.25	−22.0	−24.0	−16.0
3	Withaferin A	4.18	C_28_H_38_O_6_	470.26	471.25	299.20	−18.0	−15.0	−22.0
						67.05	−18.0	−42.0	−12.0
4	12-Deoxy-withastramonolide	5.04	C_28_H_38_O_6_	470.26	471.25	67.05	−18.0	−42.0	−12.0
						95.05	−18.0	−24.0	−18.0
5	Withanolide A	6.02	C_28_H_38_O_6_	470.26	488.30	471.25	−24.0	−13.0	−36.0
						289.20	−24.0	−23.0	−20.0
6	Withanone	6.19	C_28_H_38_O_6_	470.26	417.25	263.15	−28.0	−20.0	−28.0
						194.15	−20.0	−44.0	−20.0
7	Withanolide B	8.23	C_28_H_38_O_5_	454.27	472.30	171.15	−28.0	−36.0	−18.0
						109.15	−24.0	−40.0	−20.0
8	Fluoxymesterone	3.71	C_20_H_29_FO_3_	336.21	337.20	91.15	−10.0	−61.0	−34.0
						77.10	−10.0	−76.0	−30.0
9	Difenoconazole	8.68	C_19_H_17_Cl_2_N_3_O_3_	405.06	406.10	336.90	−12.0	−18.0	−23.0
						111.00	−12.0	−55.0	−21.0

*CE—collision energy; Q1—single quadrupole; Q3—triple quadrupole; eV—Electronvolt.

**Table 6:** Pharmacokinetic parameters for withanosides and withanolides in rats upon oral administration of the extract at a dose of 500 mg/kg.

PK Parameters	Unit	Withanoside IV	Withaferin A	12-Deoxy-Withastramonolide	Withanolide A
C*_max_*	ng/mL	13.833 ± 3.727	124.415 ± 64.932	57.536 ± 7.523	7.283 ± 3.341
T*_max_*	h	0.750 ± 0.000	0.250 ± 0.000	0.291 ± 0.102	0.333 ± 0.129
t_/1/2_	h	1.101 ± 0.272	3.148 ± 0.612	1.734 ± 0.505	0.728 ± 0.423
K*_el_*	h^−1^	0.655 ± 0.126	0.226 ± 0.038	0.436 ± 0.154	1.409 ± 1.133
AUC_(0–24)_	h.ng/mL	13.960 ± 3.703	161.180 ± 18.863	82.866 ± 7.820	4.179 ± 1.032
AUC_(0–∞)_	h.ng/mL	22.940 ± 5.730	187.645 ± 20.488	92.253 ± 13.485	7.531 ± 1.826
AUC_(0–t)/(0–inf_obs)_	-	0.611 ± 0.077	0.859 ± 0.057	0.904 ± 0.059	0.585 ± 0.201
AUMC_(0–∞)_	h.ng/mL	47.843 ± 14.715	724.870 ± 204.685	221.475 ± 92.224	10.122 ± 6.733
MRT_(0–∞)_	h.ng/mL	2.076 ± 0.394	3.846 ± 0.857	2.340 ± 0.666	1.250 ± 0.571
*Cl*/*F*	(ng/mL)/h	0.176 ± 0.037	0.026 ± 0.003	0.016 ± 0.002	0.356 ± 0.090
V*_dapp_*	(ng/mL)	0.278 ± 0.083	0.118 ± 0.023	0.040 ± 0.009	0.340 ± 0.144

Data represented as mean ± SD, (*n* = 6); C*_max_*—maximum observed concentration; T*_max_*—maximum observed time; t_1/2_—half-life; K*_el_*—elimination rate constant; AUC—area under the curve; AUMC—area under the moment curve; MRT—mean residence time; *Cl*/*F*—oral clearance; V*_dapp_*—mean apparent volume of distribution.

**Table 7:** Apparent permeability of *Withania Somnifera* extract constituents from the intestinal mucosa.

Content	Apparent Permeability (P*_app_*) *
Withanoside IV	1.4174 × 10^−7^ ± 1.80 × 10^−7^
Withanoside V	3.4254 × 10^−8^ ± 2.16 × 10^−8^
Withaferin A	1.1252 × 10^−7^ ± 7.15 × 10^−8^
12-Deoxy-withastramonolide	1.2221 × 10^−7^ ± 1.48 × 10^−7^
Withanolide A	6.6487 × 10^−8^ ± 5.09 × 10^−8^
Withanolide B	1.3065 × 10^−8^ ± 1.33 × 10^−8^
Withanone	3.1746 × 10^−8^ ± 1.71 × 10^−8^

* Data represented as mean ± SD, (*n* = 6).

## Data Availability

All the data generated in the current research work has been included in the manuscript.

## Supplementary Materials

The following supporting information can be downloaded at online: Figure S1. Typical MRM optimization with precursor ion and product ions (transitions) in the chromatogram with MS/MS spectra of seven analytes and internal standards in the developed UHPLC-MS/MS method, Table S1. The linear equation, linear range, and LLOQ of the withanoside IV, withanoside V, withaferin A, 12-Deoxy-withastramonolide, withanolide A, and withanolide B separately and in rat plasma samples, Table S2. Intra- and inter-day precision and accuracies of the analytes in rat plasma (ng/mL), Table S3. Extraction recovery (ng/mL) of the constituents in rat plasma samples, (*n* = 6), Table S4. Matrix effect (ng/mL) for the determination of constituents in rat plasma samples, (*n* = 6), Table S5. Dilution integrity of analytes in 20-fold and 6-fold dilution (ng/mL), (*n* = 6), Table S6. Stability assays for the determination of constituents in rat plasma, (*n* = 6).

## Abbreviations

AUC	Area under the Curve
AUMC	Area under the mean concentration
CPCSEA	The committee for the purpose of control and supervision of experiments on animals
CYP	Cytochrome P450
EMA	European medicines agency
HQC	Highest concentration QC sample
IS	Internal standard
KRB	Kreb’s ringer buffer
LLOQ	Lower limit of quantification
LQC	Lowest concentration QC sample
MQC	Moderate concentration QC sample
MRT	Mean residence time
Papp	Apparent permeability constant
RE	Relative error
ROP	Retro-orbital venous plexus
RSD	Relative standard deviation
TPSA	Topological polar surface area
UHPLC	Ultra-performance liquid chromatography
USFDA	United States food and drug administration
USP	United States pharmacopeia
WSE	*Withania somnifera* extract
