# Supplementary material for: Pharmacokinetic Study of Withanosides and Withanolides from Withania somnifera Using Ultra-High Performance Liquid Chromatography-Tandem Mass Spectrometry (UHPLC-MS/MS)
Source: Molecules. 2022 Feb 22;27(5):1476. doi: 10.3390/molecules27051476 (PMC8912008; doi:10.3390/molecules27051476)
Supplement: Supplementary file 1 [file molecules-27-01476-s001.zip › molecules-1553137-supplementary.pdf]

Pharmacokinetic Study of Withanosides and Withanolides from *Withania somnifera* using Ultra-High Performance Liquid Chromatography-Tandem Mass Spectrometry (UHPLC-MS/MS)

Siddharth J. Modi[1,2#], Anshuly Tiwari[1#], Chetana Ghule[1#], Sandeep Pawar[1#], Shubham Jagtap[1], Ganesh Saste[1], Ruchi Singh[2], Amol Deshmukh[3], Aboli Girme[1*], and Lal Hingorani[1,2,3]

[1]Analytical Development and Innovation Center, Pharmanza Herbal Pvt. Ltd., Anand-388435, Gujarat, India.

[2]New Product Development Department, Pharmanza Herbal Pvt. Ltd., Anand-388435, Gujarat, India.

[3]Clinical Research and Intellectual Property Rights, Pharmanza Herbal Pvt. Ltd., Anand-388435, Gujarat, India.

[#]Authors contributed equally.

***Corresponding author:** Dr Aboli Girme, Pharmanza Herbal Pvt. Ltd, Address- Plot # 214, Borsad-Tarapur Road, Nr. Vadadla Patiya, At & PO: Kaniya-388435, Tal: Petlad, Dist: Anand (Gujarat) India. Tel.: +91-7043534016/+91-9825063959. E-mail: ardm@pharmanzaherbals.com

Supplementary Tables

Table S1. The linear equation, linear range, and LLOQ of the withanoside IV, withanoside V, withaferin A, 12-Deoxy-withastramonolide, withanolide A, withanolide B, and withanone in rat plasma samples.

Table S2. Intra- and inter-day precision and accuracies of the analytes in rat plasma samples, (ng/mL).

Table S3. Extraction recovery (ng/mL) of the constituents in rat plasma samples, ($n = 6$).

Table S4. Matrix effect (ng/mL) for the determination of constituents in rat plasma samples, ($n = 6$).

Table S5. Dilution integrity of analytes in 20-fold and 6-fold dilution (ng/mL), ($n = 6$).

Table S6. Stability assays for the determination of constituents in rat plasma samples, ($n = 6$).

Table S1. The linear equation, linear range, and LLOQ of the withanoside IV, withanoside V, withaferin A, 12-Deoxy-withastramonolide, withanolide A, withanolide B, and withanone in rat plasma samples.

Analyte	Linear equation	Range (ng/mL)	r^2	LLOQ (ng/mL)
**WSIV**	$y = 0.8519229x + 0.02379023$	3.00 to 400.00	0.9976	3.00
**WSV**	$y = 2.828480x + 0.04256550$	3.00 to 400.00	0.9917	3.00
**WFA**	$y = 5.841658x + 0.1158392$	3.00 to 400.00	0.9958	3.00
**WSL**	$y = 2751826x + 0.03420022$	3.00 to 400.00	0.9972	3.00
**WLA**	$y = 1.731572x + 0.02476672$	3.00 to 400.00	0.9949	3.00
**WLB**	$y = 3.981638x + 0.02096910$	3.00 to 400.00	0.9946	3.00
**WNO**	$y = 15.34367x + 0.06067664$	3.00 to 400.00	0.9971	3.00

*WSIV-withanoside IV; WSV-withanoside V; WFA- withaferin A; WSL-12-Deoxy-withastramonolide; WLA-withanolide A; WLB-withanolide B; WNO-withanone; r^2-correlation coefficient; LLOQ-lower limit of quantification.

Table S2. Intra and inter-day precision and accuracies of the analytes in rat plasma samples, (ng/mL).

Analyte	Nominal concentration (ng/mL)	Intra-day 01 (*n* = 6)		Intra-day 02 (*n* = 6)		Intra-day 03 (*n* = 6)		Inter-day (*n* = 6x3)	
		Precision (RSD, %)	Accuracy (RE, %)	Precision (RSD, %)	Accuracy (RE, %)	Precision (RSD, %)	Accuracy (RE, %)	Precision (RSD, %)	Accuracy (RE, %)
**WSIV**	3.00	6.17	7.94	7.34	1.39	4.21	10.78	4.51	6.67
	10.05	3.81	7.35	2.36	-7.97	1.83	8.20	8.91	2.53
	75.50	4.21	-7.94	4.25	-0.82	1.64	9.70	8.85	0.31
	200.50	3.66	4.25	3.82	0.69	0.79	11.61	5.28	5.52
**WSV**	3.00	3.48	-2.61	3.36	-13.00	3.19	10.22	11.90	-1.78
	10.05	4.19	-3.77	6.33	-1.88	7.59	9.82	7.27	1.37
	75.50	2.83	1.87	3.84	2.17	4.44	5.22	1.80	3.09
	200.50	3.86	4.15	3.93	-1.95	4.08	11.25	6.33	4.49
**WFA**	3.00	4.42	-4.17	8.58	-0.89	5.00	3.67	3.88	-0.44
	10.05	6.42	-3.92	7.57	-3.35	4.79	3.08	3.93	-1.37
	75.50	2.62	5.79	3.30	-5.58	3.64	5.33	6.32	1.85
	200.50	2.14	-6.25	2.76	-6.24	1.47	8.40	8.57	-1.37
**WSL**	3.30	3.25	0.38	8.26	-5.28	7.33	1.00	3.53	-1.33
	10.50	8.88	1.62	6.11	-8.68	3.18	1.73	6.09	-1.80
	76.00	5.44	9.06	2.21	-4.46	2.50	4.16	6.65	2.92
	201.50	3.03	-4.22	4.49	-5.39	1.79	8.67	7.82	-0.31
**WLA**	3.00	5.70	-6.61	7.02	-12.83	7.20	0.44	6.95	-6.33
	10.20	8.45	0.05	7.71	-1.88	3.64	5.35	3.73	1.20
	76.00	7.33	2.31	7.80	-1.07	2.67	4.76	2.87	2.00
	201.50	1.96	0.36	3.70	-2.20	3.46	5.04	3.63	1.07
**WLB**	3.00	4.55	1.28	7.53	5.28	5.13	0.11	2.72	2.22
	10.05	7.71	-3.55	4.96	-0.97	4.59	-1.13	0.32	-0.87
	75.50	5.29	-3.08	3.04	-5.28	2.96	-6.51	1.83	-4.96
	200.50	3.34	4.97	2.88	-9.04	3.46	-2.74	7.18	-2.27
**WNO**	3.06	5.52	-4.72	6.67	2.78	1.79	6.00	5.39	1.33
	10.20	7.76	-0.70	4.65	-2.23	3.66	9.47	6.23	2.20
	76.00	2.75	1.72	2.87	-5.14	4.03	5.57	5.38	0.72
	201.50	3.30	-3.83	2.79	-8.34	4.37	11.25	10.29	-0.31

* WSIV-withanoside IV; WSV-withanoside V; WFA- withaferin A; WSL-12-Deoxy-withastramonolide; WLA-withanolide A; WLB-withanolide B; WNO-withanone; RSD-relative standard deviation; RE-relative error.

Table S3. Extraction recovery (ng/mL) of the constituents in rat plasma samples, (*n* = *6*).

Analyte	Nominal concentration (ng/mL)	Extraction recovery (% ± SD)	Precision (RSD, %)	Accuracy (RE, %)
**WSIV**	10.05	95.38±3.64	2.49	6.58
	75.50	92.06±3.87	2.96	-0.90
	200.50	99.84±1.23	3.66	4.25
**WSV**	10.05	96.25±4.02	5.03	-0.87
	75.50	99.94±1.84	2.47	3.19
	200.50	99.67±5.07	3.25	-4.21
**WFA**	10.05	96.10±6.16	4.78	-5.80
	75.50	99.96±2.73	0.64	-1.97
	200.50	93.75±2.00	1.34	-1.91
**WSL**	10.50	98.28±4.76	8.15	0.98
	76.00	99.92±4.04	4.42	-0.90
	201.50	95.78±2.90	1.19	-1.19
**WLA**	10.20	96.73±6.67	5.72	1.35
	76.00	98.94±5.12	3.39	5.15
	201.50	98.96±2.85	2.80	-1.05
**WLB**	10.05	96.45±7.46	5.22	7.93
	75.50	96.92±5.13	4.93	2.18
	200.50	99.60±2.91	4.90	-0.55
**WNO**	10.20	99.29±7.70	3.30	-3.15
	76.00	99.71±1.63	2.08	-0.19
	201.50	96.16±3.17	1.60	-1.34

* WSIV-withanoside IV; WSV-withanoside V; WFA- withaferin A; WSL-12-Deoxy-withastramonolide; WLA-withanolide A; WLB-withanolide B; WNO-withanone; SD-standard deviation; RSD-relative standard deviation; RE-relative error.

Table S4. Matrix effect (ng/mL) for the determination of constituents in rat plasma samples, (n = 6).

Analyte	Nominal Concentration (ng/mL)	Matrix Effect (% ± SD)	Precision (RSD, %)	Accuracy (RE, %)
**WSIV**	10.05	85.61 ± 2.79	3.26	11.05
	200.50	92.15 ± 3.85	4.18	9.30
**WSV**	10.05	103.32 ± 4.14	4.01	9.47
	200.50	100.71 ± 5.09	5.06	6.79
**WFA**	10.05	87.96 ± 8.74	9.93	10.12
	200.50	85.28 ± 2.16	2.53	11.35
**WSL**	10.50	102.18 ± 3.62	3.54	7.47
	201.50	101.51 ± 1.87	1.84	8.93
**WLA**	10.20	97.53 ± 13.21	13.55	1.28
	201.50	88.43 ± 3.91	4.42	10.63
**WLB**	10.05	109.77 ± 8.46	7.71	-9.50
	200.50	95.96 ± 4.88	5.08	-5.12
**WNO**	10.20	105.61 ± 5.60	5.30	-7.25
	201.50	86.12 ± 2.22	2.57	7.52

* WSIV-withanoside IV; WSV-withanoside V; WFA- withaferin A; WSL-12-Deoxy-withastramonolide; WLA-withanolide A; WLB-withanolide B; WNO-withanone; SD-standard deviation; RSD-relative standard deviation; RE-relative error.

Table S5. Dilution integrity of analytes in 20-fold and 6-fold dilution (ng/mL), (*n* = *6*).

Analyte	Nominal Concentration (ng/mL)	20-Fold Dilution (ng/mL)	Precision (RSD, %)	Accuracy (RE, %)	6-Fold Dilution (ng/mL)	Precisio n (RSD, %)	Accuracy (RE, %)
**WSIV**	450.00	22.85±0.48	2.10	-8.58	66.60±3.04	4.56	-11.20
**WSV**		22.51±1.14	5.08	-9.94	66.41±1.60	2.41	-11.46
**WFA**		27.41±0.41	1.50	9.62	76.01±2.33	3.07	1.35
**WSL**		27.24±0.39	1.44	8.97	72.03±2.94	4.08	-3.96
**WLA**		22.49±0.76	3.39	-10.04	67.05±1.25	1.86	-10.6
**WLB**		27.64±0.52	1.87	10.58	79.37±1.70	2.15	5.82
**WNO**		27.01±0.86	3.18	8.04	71.73±1.98	2.76	-4.36

* WSIV-withanoside IV; WSV-withanoside V; WFA- withaferin A; WSL-12-Deoxy-withastramonolide; WLA-withanolide A; WLB-withanolide B; WNO-withanone RSD-relative standard deviation; RE-relative error.

Table S6. Stability assays for the determination of constituents in rat plasma samples, (*n = 6*).

Analyte	Nominal concentration (ng/mL)	Autosampler stability (% ± SD)	Precision (RSD, %)	Accuracy (RE, %)	Freeze-thaw stability (% ± SD)	Precision (RSD, %)	Accuracy (RE, %)	Bench top Stability (% ± SD)	Precision (RSD, %)	Accuracy (RE, %)	Processed sample stability (% ± SD)	Precision (RSD, %)	Accuracy (RE, %)	Long-term stability (% ± SD)	Precision (RSD, %)	Accuracy (RE, %)
**WSIV**	10.05	108.21 ± 2.00	1.47	-0.59	104.78 ± 5.59	5.33	4.78	104.41 ± 4.84	5.91	6.70	104.41 ± 4.84	5.22	6.02	10.21 ± 0.62	6.03	2.10
	200.50	111.61 ± 0.88	3.48	-2.19	98.95 ± 5.47	5.53	-1.05	100.34 ± 10.45	7.76	-1.94	101.10 ± 9.54	8.09	-0.51	192.17 ± 11.10	5.78	-3.92
**WSV**	10.05	109.83 ± 8.35	6.15	11.78	105.13 ± 6.95	6.60	5.13	99.58 ± 8.68	10.05	0.60	134.85 ± 90.10	9.84	-0.88	9.28 ± 0.25	2.66	-7.22
	200.50	111.25 ± 4.54	3.22	10.61	99.38 ± 6.58	6.62	-0.62	91.74 ± 8.97	6.26	-9.28	90.47 ± 6.50	6.22	-9.07	183.24 ± 10.63	5.80	-8.38
**WFA**	10.05	103.09 ± 4.95	6.70	-0.93	103.41 ± 5.62	5.42	3.41	103.30 ± 8.41	8.02	-2.78	103.30 ± 8.41	6.14	1.20	9.49 ± 0.58	6.16	-5.12
	200.50	108.40 ± 1.59	1.02	8.88	102.10 ± 5.76	5.64	2.10	94.72 ± 6.57	9.39	0.81	99.56 ± 5.11	8.00	1.68	204.07± 8.81	4.32	2.03
**WSL**	10.50	101.72 ± 3.25	6.90	-2.75	101.34 ± 8.37	8.25	1.34	97.92 ± 7.04	8.93	-4.53	121.14 ± 59.07	9.18	-5.15	9.46 ± 0.73	7.74	-5.4
	201.50	108.67 ± 1.94	1.53	8.57	97.78 ± 4.28	4.37	-2.22	95.97 ± 7.13	5.03	-1.58	92.99 ± 8.83	8.41	-3.94	188.50 ± 5.37	2.85	-5.75
**WLA**	10.20	105.35 ± 3.83	3.16	8.55	102.18 ± 6.01	5.88	2.19	102.00 ± 7.99	7.00	4.57	128.43 ± 68.06	8.04	2.98	10.48 ± 0.59	5.63	4.78
	201.50	105.04 ± 3.63	3.65	8.11	98.89 ± 4.24	4.29	-1.11	103.63 ± 6.10	6.41	1.06	99.36 ± 6.79	5.82	-1.62	194.28 ± 5.94	3.06	-2.86
**WLB**	10.05	98.84 ± 4.54	6.39	-2.65	116.31 ± 36.76	6.43	-0.12	95.51 ± 5.04	6.21	-5.15	113.48 ± 47.73	5.88	-2.88	9.50 ± 0.55	5.76	-5.03
	200.50	97.26 ± 3.36	6.22	4.36	93.48 ± 6.55	7.00	-6.52	96.83 ± 6.66	5.78	-2.52	95.01 ± 5.83	4.61	-6.61	189.63 ± 5.10	2.69	-5.19
**WNO**	10.20	109.47 ± 4.01	7.81	3.48	104.24 ± 7.98	7.81	3.00	96.31 ± 6.27	10.95	-2.70	112.07 ± 37.93	6.53	-1.47	9.76 ± 0.69	7.09	-2.37
	201.50	111.25 ± 4.86	4.4	8.64	98.14 ± 4.74	4.83	-1.86	98.87 ± 9.47	4.53	-2.12	99.56 ± 4.91	5.37	-2.67	194.31 ± 8.31	4.28	-2.84

* WSIV-withanoside IV; WSV-withanoside V; WFA- withaferin A; WSL-12-Deoxy-withastramonolide; WLA-withanolide A; WLB-withanolide B; WNO-withanone; SD-standard deviation; RSD-relative standard deviation; RE-relative error.

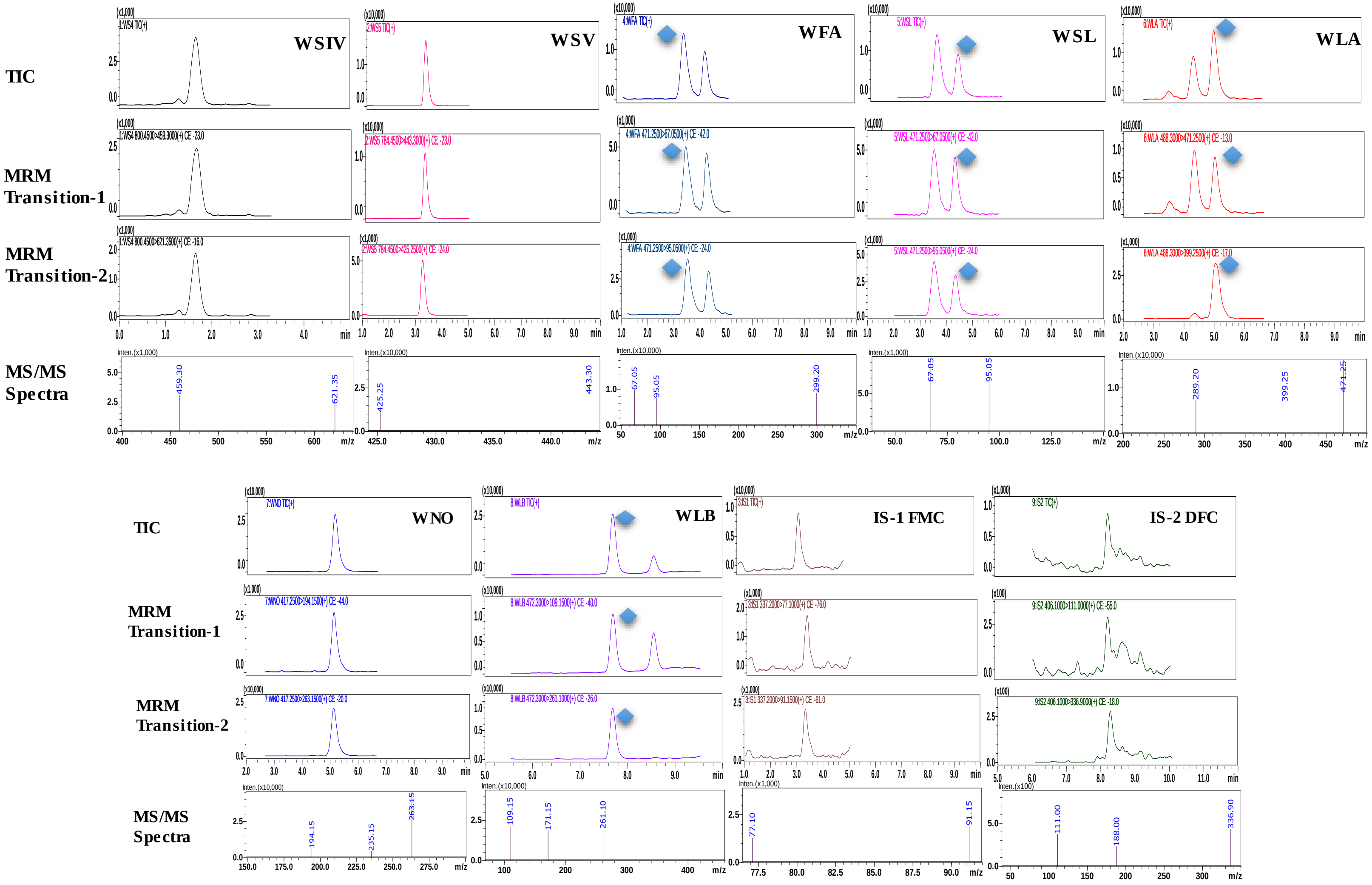

Figure S1. Typical MRM optimization with precursor ion and product ions (transitions) in the chromatogram with MS/MS spectra of seven WS analytes and internal standards in the developed UHPLC-MS/MS method.
